# Supplementary material for: A Novel UHPLC-MS/MS Based Method for Isomeric Separation and Quantitative Determination of Cyanogenic Glycosides in American Elderberry
Source: Metabolites. 2024 Jun 26;14(7):360. doi: 10.3390/metabo14070360 (PMC11279188; doi:10.3390/metabo14070360)
Supplement: Supplementary file 1 [file metabolites-14-00360-s001.zip › metabolites-3049250-supplementary.pdf]

metabolites

MDPI

Supporting Information

Article

A Novel UHPLC-MS/MS based method for isomeric separation and quantitative determination of cyanogenic glycosides in American elderberry samples

Deepak M. Kasote [1,†], Zhentian Lei [1,2,*], Clayton D. Kranawetter [2], Ashley Conway-Anderson [3], Barbara W. Sumner [1] and Lloyd W. Sumner [1,2,*]

1 Metabolomics Center, University of Missouri-Columbia, Columbia, MO 65211, USA; deepakkasote06@gmail.com (D.M.K.); sumnerb@missouri.edu (B.W.S.)
2 Department of Biochemistry, University of Missouri-Columbia, Columbia, MO 65211, USA; cdk374@mail.missouri.edu
3 Center for Agroforestry, University of Missouri-Columbia, Columbia, MO 65211, USA; acconway@missouri.edu
* Correspondence: leiz@missouri.edu (Z.L.); sumnerlw@missouri.edu (L.W.S.)
† Current affiliation: Agricultural Research Station, Qatar University, Doha P.O. Box 2713, Qatar.

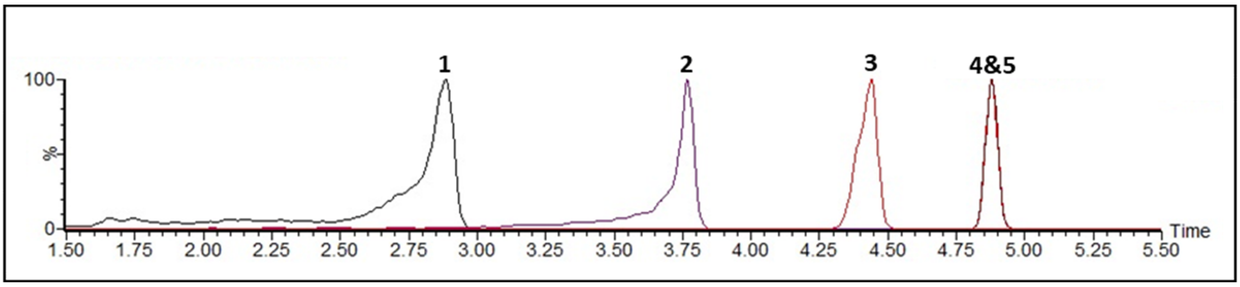

Figure S1. MRM chromatogram of cyanogenic glucosides standards, 1. Linamarin, 2. Dhurrin, 3. Amygdalin, 4. (*R*)-Prunasin and 5. (*S*)-Prunasin (Sambunigrin) using literature-reported gradient elution program [10].

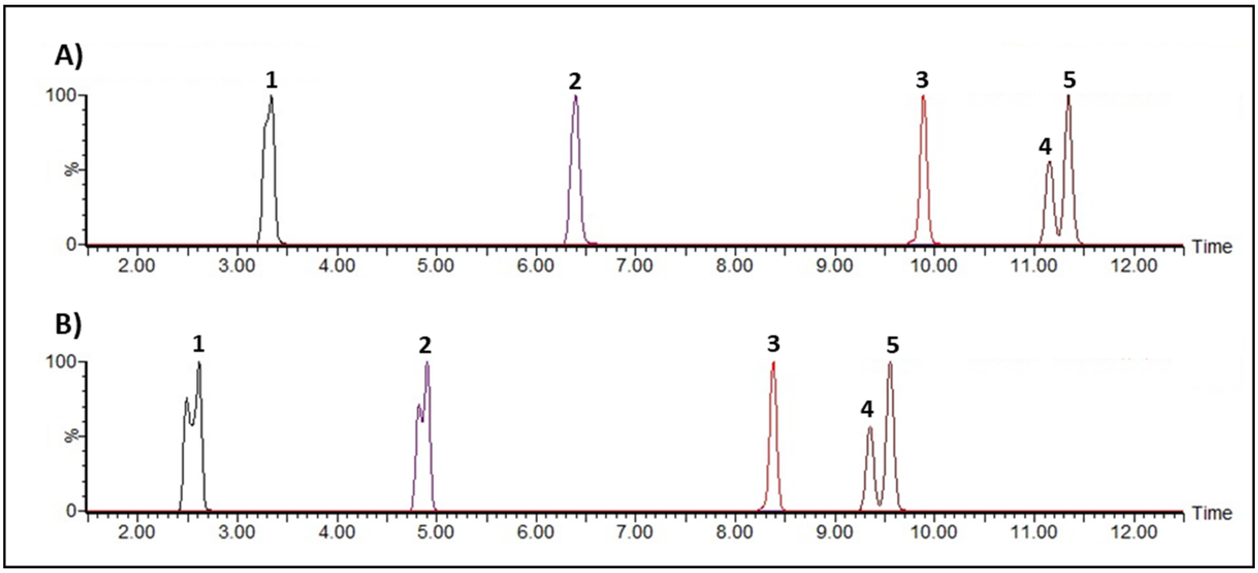

Figure S2. Effect of column type on separation efficiencies of cyanogenic glucosides standards, 1. Linamarin, 2. Dhurrin, 3. Amygdalin, 4. (*R*)-Prunasin and 5. (*S*)-Prunasin (Sambunigrin). A) ACQUITY UPLC HSS T3 column (1.8 uM, 2.1 × 100 mm). B) and ACQUITY Premier BEHTM C18 column (1.7 µm, 2.1 × 100 mm).

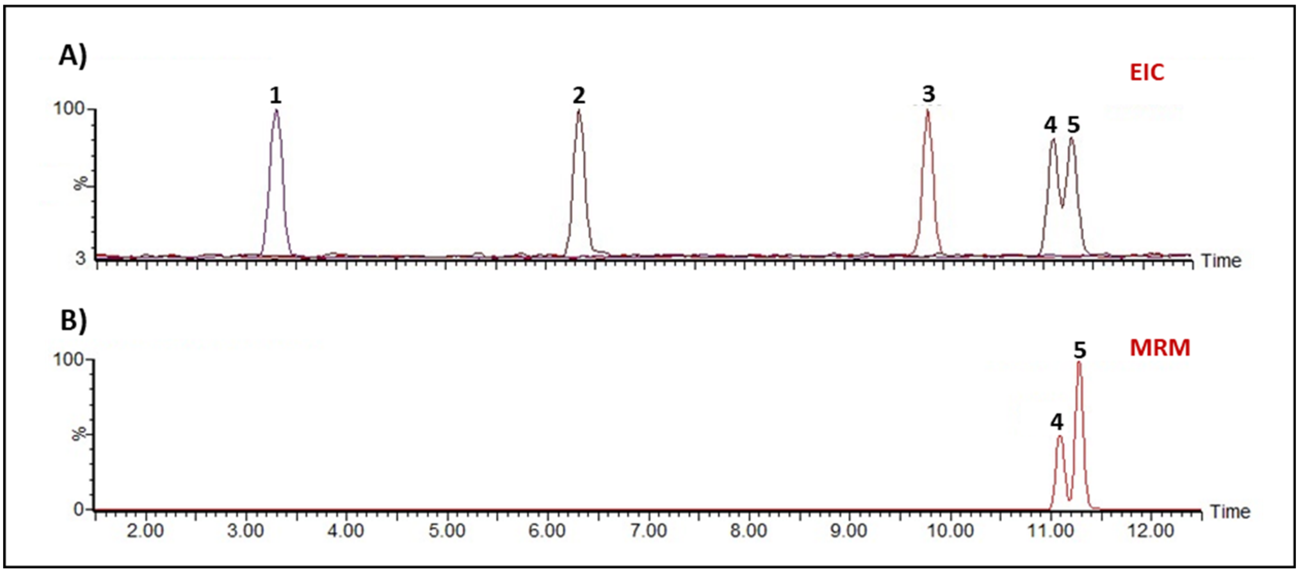

Figure S3. A) Extracted ion chromatogram (EIC) of sodium $[M+Na]^+$ adducts of a CNGs standard mixture after using aqueous mobile phases containing 0.1% formic acid. B) Multiple reaction monitoring (MRM) chromatogram $[M+Na]^+$ adducts of a CNGs standard mixture after using aqueous mobile phases containing 0.1% formic acid. 1. Linamarin, 2. Dhurrin, 3. Amygdalin, 4. (*R*)-Prunasin and 5. (*S*)-Prunasin (Sambunigrin)

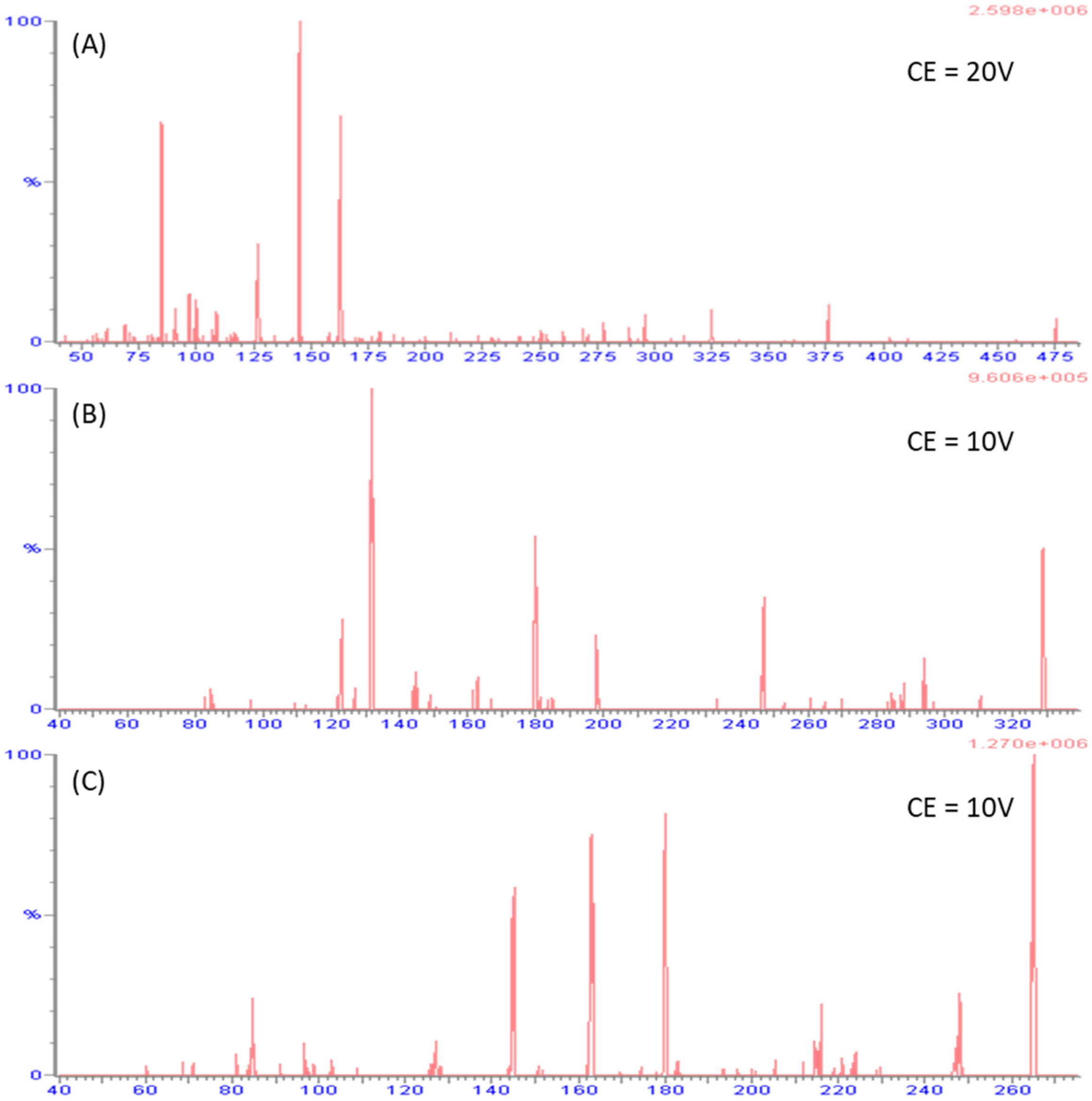

Figure S4. Mass fragmentation spectra of amygdalin (A), dhurrin (B), and linamarin (C) at ESI(+) mode.

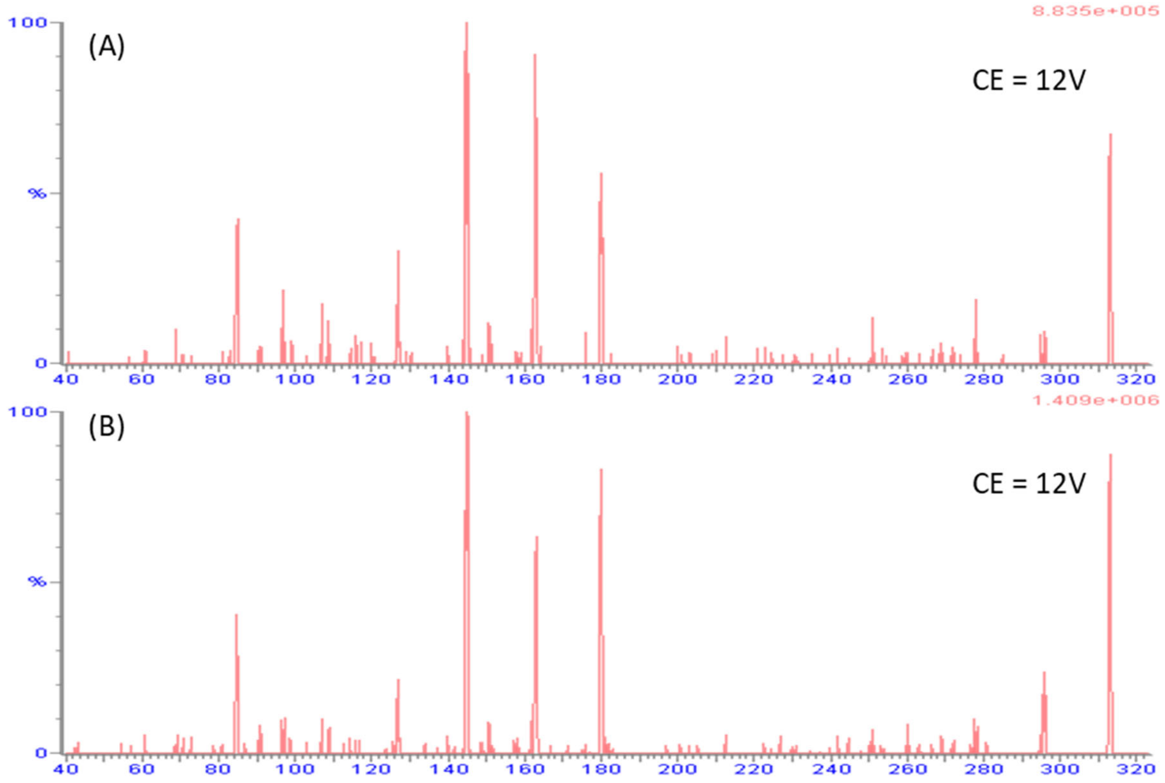

Figure S5. Mass fragmentation spectra of (*R*)-prunasin and (*S*)-prunasin (sambunigrin) isomers at ESI(+) mode.

Table S1. Transitions used for the analysis of sodium $[M+Na]^+$ adducts of cyanogenic glucosides standards.

Sr. No.	Compound	Transition (m/z)	Cone (V)	Collision (eV)
1.	Linamarin	270.11 > 213.77	4	10
		270.11 > 117.95	4	8
2.	Dhurrin	333.94 > 214.15	8	26
		333.94 > 118.06	8	10
3.	Amygdalin	479.88 > 214.15	24	30
		479.88 > 118.01	24	20
4.	(*R*)-Prunasin	317.87 > 231.96	14	8
		317.87 > 117.96	14	12
5.	(*S*)-Prunasin (Sambunigrin)	317.87 > 231.96	14	8
		317.87 > 117.96	14	12

Table S2. Matrix effect before and after solid-phase extraction (SPE) in different matrices of elderberry.

Sr. No.	Compound	Before SPE		After SPE	
		Leaf tissue	Fruit tissue	Leaf tissue	Fruit tissue
1.	Linamarin	-23.6	1.2	-19.9	11.5
2.	Dhurrin	45.6	7.3	26.4	11.8
3.	Amygdalin	32.4	9.2	17.2	17.8
4.	(*R*)-Prunasin	8.3	13.1	36.6	28.7
5.	(*S*)-prunasin (Sambunigrin)	31.4	14.9	26.7	21
